# Exploiting Zebrafish Xenografts for Testing the *in vivo* Antitumorigenic Activity of Microcin E492 Against Human Colorectal Cancer Cells

**DOI:** 10.3389/fmicb.2020.00405

**Published:** 2020-03-19

**Authors:** Macarena A. Varas, Carlos Muñoz-Montecinos, Violeta Kallens, Valeska Simon, Miguel L. Allende, Andrés E. Marcoleta, Rosalba Lagos

**Affiliations:** ^1^Laboratorio de Biología Estructural y Molecular BEM, Departamento de Biología, Facultad de Ciencias, Universidad de Chile, Santiago, Chile; ^2^Departamento de Biología, Facultad de Ciencias, FONDAP Center for Genome Regulation, Universidad de Chile, Santiago, Chile; ^3^Laboratorio de Inmunología, Departamento de Biología, Facultad de Ciencias, Universidad de Chile, Santiago, Chile

**Keywords:** microcin E492, antitumorigenic peptide, zebrafish xenograft, bacteriocin, colorectal cancer, human cell lines, cytotoxicity, *Klebsiella pneumoniae*

## Abstract

One of the approaches to address cancer treatment is to develop new drugs not only to obtain compounds with less side effects, but also to have a broader set of alternatives to tackle the resistant forms of this pathology. In this regard, growing evidence supports the use of bacteria-derived peptides such as bacteriocins, which have emerged as promising anti-cancer molecules. In addition to test the activity of these molecules on cancer cells in culture, their *in vivo* antitumorigenic properties must be validated in animal models. Although the standard approach for such assays employs experiments in nude mice, at the initial stages of testing, the use of high-throughput animal models would permit rapid proof-of-concept experiments, screening a high number of compounds, and thus increasing the possibilities of finding new anti-cancer molecules. A validated and promising alternative animal model are zebrafish larvae harboring xenografts of human cancer cells. Here, we addressed the anti-cancer properties of the antibacterial peptide microcin E492 (MccE492), a bacteriocin produced by *Klebsiella pneumoniae*, showing that this peptide has a marked cytotoxic effect on human colorectal cancer cells *in vitro*. Furthermore, we developed a zebrafish xenograft model using these cells to test the antitumor effect of MccE492 *in vivo*, demonstrating that intratumor injection of this peptide significantly reduced the tumor cell mass. Our results provide, for the first time, evidence of the *in vivo* antitumoral properties of a bacteriocin tested in an animal model. This evidence strongly supports the potential of this bacteriocin for the development of novel anti-cancer therapies.

## Introduction

**Figure 1:**
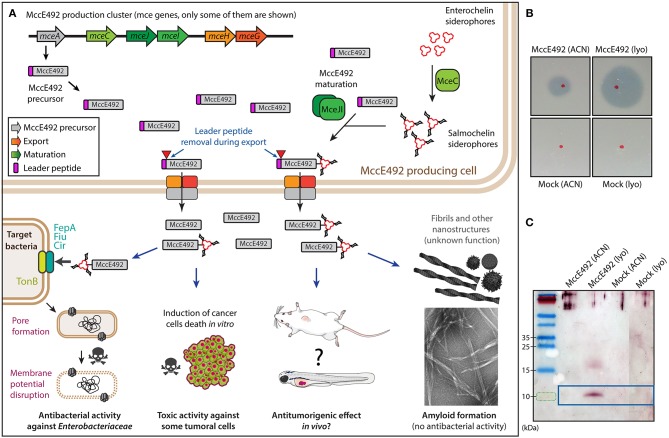
**(A)** Main features of microcin E492 peptide and its production by bacterial cells, through the expression of the *mce* genes from the MccE492 gene cluster. The *mceA* gene encodes the microcin precursor that includes a leader peptide that is removed during the export by ABC exporters encoded by *mceHG*. During maturation, salmochelin (a glycosylated enterochelin derivative synthetized by MceC) is attached to MccE492 C-terminus by MceIJ. Mature MccE492 is recognized and internalized by catechol siderophore receptors of the target bacterial cell, and once in the periplasm it inserts into the cytoplasmic membrane in a TonB-dependent process forming pores that lead to a toxic effect through the disruption of the membrane potential of the cell. Additionally, under certain conditions, MccE492 aggregates into amyloid fibers and rod-shaped nanostructures, leading to the loss of its antibacterial activity. MccE492 also has toxic activity *in vitro* against some tumor cell lines, although its *in vivo* antitumoral effect has not been validated in animal models. For simplicity, several genes from the MccE492 gene cluster are not included, among them the immunity gene *mceB* that is encoded in an operon with the structural gene *mceA*. **(B)** Antibacterial activity assay of a representative MccE492 preparation (either the ACN fraction or after lyophilization) assessed through the detection of growth inhibition halos over sensitive bacterial lawns. The red dots correspond to the zone of the lawn where the drops of MccE492 or the mock preparation were placed. **(C)** SDS-PAGE and immunoblot of a representative MccE492 preparation (either the ACN fraction or after lyophilization), revealed with a monoclonal anti-McccE492 antibody. The last lane of the immunoblot image (showing mock purification) was spliced together with the first 3 lanes, to avoid showing blank (non-loaded) lanes.

Cancer is one of the leading causes of death in the world and, for this reason, research focused on the search of new agents that inhibit the proliferation of tumors is highly relevant. A promising area of research involves the study of therapeutic peptides and proteins produced by bacteria, including bacteriocins. Bacteriocins, besides their characteristic antibacterial properties, have a potential role as anticancer agents. The use of bacteria or their products against cancer has already been tested at different levels. For instance, a treatment with mixed extracts from *Streptococcus pyogenes* and *Serratia marscensces* (currently known as Coley toxins) on unresectable tumors was successfully administrated more than 100 years ago (Karpiński and Adamczak, 2018; reviewed in Baindara et al., 2018; Ashu et al., 2019). Similarly, the study of different bacteriocins and their antineoplasic properties has a long history and has included colicins, pyocins, pediocins, and microcins (reviewed in Lagos, 2007; Cornut et al., 2008). Over the past few years there has been a renewed interest in exploring anticancer properties of other bacteriocins, and among them are laterosporulin10 (Baindara et al., 2017), nisin ZP (Kamarajan et al., 2015), nisin (Joo et al., 2012), plantaricin P1053 (De Giani et al., 2019), and others (reviewed in Kaur and Kaur, 2015; Baindara et al., 2018; Karpiński and Adamczak, 2018; Ashu et al., 2019). Microcins are a class of bacteriocins with a molecular mass of <10 kDa produced by Gram-negative bacteria, principally *Enterobactericeae*. A previous study on microcin E492 (MccE492) reported on its capacity to inhibit the proliferation of cancer cells *in vitro* (Hetz et al., 2002; Lagos et al., 2009). MccE492 is a pore-forming bacteriocin displaying toxic activity on several strains of the family *Enterobacteriaceae* (de Lorenzo and Pugsley, 1985; Lagos et al., 1993, 2001). This bacteriocin was isolated from *Klebsiella pneumoniae* RYC492 (de Lorenzo, 1984), and the genetic determinants necessary for its production were cloned and expressed in *Escherichia coli* (Wilkens et al., 1997). The steps for MccE92 synthesis, post-translational modification, processing, export and uptake from target cells are shown in Figure 1A (Thomas et al., 2004; Strahsburger et al., 2005; Lagos et al., 2009; Marcoleta et al., 2013a). The gene cluster encoding for active MccE492 is located in the genomic island GI-E492 (Marcoleta et al., 2013b, 2016), which was found to be highly prevalent among liver abscess-associated strains of *K. pneumoniae* (Marcoleta et al., 2016, 2018; Lam et al., 2018). A striking characteristic of MccE492 is that it forms amyloid fibers both *in vivo* and *in vitro* (Figure 1A; Bieler et al., 2005; Arranz et al., 2012; Marcoleta et al., 2013a; Aguilera et al., 2016). Regarding its cytotoxic activity, MccE492 was toxic to different malignant cell lines (HeLa, Jurkat, and RJ2.25), and did not have effect on KG-1 and a primary culture from human tonsils (Hetz et al., 2002). This study, however, did not include malignant cell lines derived from intestinal cancer, which could be a relevant target since MccE492 is produced by *K. pneumoniae*, known to colonize the intestine. To explore its potential use as an antitumor, the toxic activity of this bacteriocin should be tested *in vivo* in an animal model. The gold standard for such assays are nude mice; however, these studies are lengthy and costly for exploratory and proof-of-concept approaches. To overcome these drawbacks, the use of zebrafish as an alternative model for pre-clinical anti-cancer drug tests has gained importance (Wertman et al., 2016; Wyatt et al., 2017; Hill et al., 2018; Letrado et al., 2018; Hason and Bartůněk, 2019). This animal model has multiple advantages, among them, the possibility of concurrently examining a large number of individuals (allowing more statistically robust analyses), the duration of the experiments is brief, and a high number of experiments can be conducted at an affordable cost. Furthermore, the use of zebrafish larvae has allowed the development of human xenograft tumors showing histological features and gene expression patterns similar to those of the original human tumor. Another advantage is the optical transparency of the larvae, which allows real time tracking of individual cancer cells and tumor growth evaluation by non-invasive imaging, without the need to fix and sacrifice the animals (reviewed in White et al., 2013). Currently, zebrafish is being used for xenotransplantation of human cancers to evaluate the drug response of tumors from patient-derived biopsy specimens (reviewed in Veinotte et al., 2014). Fior et al. (2017), testing different colorectal cancers, demonstrated the usefulness of this model to predict the response of a specific cancer to a given therapy using patient-derived xenografts in zebrafish. These findings support the use of this model to test bacteriocins as anticancer compounds in colorectal tumors. Preliminary but not conclusive results from our laboratory (Lagos et al., 2009) suggested the possible antitumoral activity of McccE492 tested on tumors derived from colorectal cancer cells developed in nude mice. To demonstrate if indeed MccE492 has antitumor capacity *in vivo*, we decided to exploit the zebrafish model. Here, we tested the effect of MccE492 on two human colorectal cancer cell lines *in vitro*, and used one of them to develop a zebrafish xenograft model to assess the effect of direct MccE492 intratumoral injection on tumor growth.

## Materials and Methods

### Microcin E492 Purification

*Escherichia coli* BL21 DE3 cells were transformed with the pMccE492 plasmid carrying a 13-kbp segment of the microcin E492 production cluster, originally cloned from *Klebsiella pneumoniae* RYC492 (Aguilera et al., 2016). An overnight culture of these cells grown in LB broth (10 g/L tryptone, 10 g/L NaCl and 5 g/L yeast extract) were typically used to inoculate (1:1000) 2 L of M9 medium supplemented with citrate and glucose (2 g/L each) and ampicillin (100 μg/mL). Cells were grown at 37°C with shaking (180 rpm) during 12–14 h, and then the supernatant was collected by centrifugation at 6,500 × g during 30 min at 4°C. Then, the supernatant was filtered through a 0.22 μm polyethersulfone membrane (Steritop, Millipore). The cell-free medium was incubated at 4°C during 2 h with 10 g of Bondapak C18 resin (Waters) previously activated overnight in 100% acetonitrile (ACN). The resin was filtered by negative pressure through a Buchner funnel, washed with 200 mL of 40% methanol, then with 200 mL of 25% ACN, and finally eluted with a 30–100% ACN stepwise gradient in fractions of 50 mL each. MccE492-enriched fractions were dialyzed twice for 2 h against 40 volumes of nanopure water and then lyophilized and stored at −20°C. For mock purification control, BL21 (DE3) cells were transformed with pJ053 plasmid, a pMccE492 derivative lacking the genes *mceA* (encoding the MccE492 precursor) and *mceB* (encoding MccE492 immunity protein). The downstream purification procedure was the same than for MccE492 purification.

### MccE492 Activity Assay on Plates

Antibacterial activity determination in plates was performed mixing an aliquot of 0.3 mL of ~1 × 10^7^ cells/mL of the *E. coli* indicator strain BL21 (DE3) with 3 mL of LB soft agar (0.7% w/v agar), and overlaying onto LB agar plates. Then, 3 μL drops of MccE492 preparations were spotted onto the top agar and incubated overnight at 37°C. Antibacterial activity was detected by the formation of growth inhibition halos.

### SDS-PAGE and Immunoblotting

Sodium dodecyl sulfate-polyacrylamide gel electrophoresis (SDS-PAGE) was performed as described by Schägger and von Jagow (1987). Nitrocellulose membranes (Millipore) were used for immunoblot transfer (1 h, 350 mA, using chilled 25 mM Tris-HCl, 190 mM glycine, 20% methanol, as the transfer buffer). MccE492 was detected with a mouse monoclonal antibody raised against a synthetic MccE492 peptide (antiserum dilution, 1:1,000; Genscript) and with a goat anti-mouse alkaline phosphatase-conjugated secondary antibody (dilution 1:2,500). The alkaline phosphatase colorimetric reaction was performed as described by Marcoleta et al. (2013a). Briefly, the membrane was washed with FAL buffer (100 mM Tris-HCl [pH 9.5], 100 mM NaCl, 5 mM MgCl_2_) and incubated in 10 ml of a mixture of BCIP (5-bromo-4-chloro-3′-indolylphosphate p-toluidine salt) and NBT (nitroblue tetrazolium chloride; 0.3 and 0.15 mg/mL in FAL buffer, respectively), until an optimal signal was observed.

### Cancer Cell Lines and Culture Conditions

The human colorectal adenocarcinoma cell lines with epithelial morphology HT29 and SW620 were directly acquired from the European Collection of Authenticated Cell Cultures (Catalog No. 91072201 and 87051203, respectively). Cells lines were maintained in 75 cm^2^ flasks (T75) at 37°C and 5% CO_2_ in Iscove's Modified Dulbecco's medium (IMDM) or RPMI 1640 medium supplemented with 10% fetal calf serum (FCS).

### Viability Assays

SW620 and HT29 cells were seeded at 2.5 × 10^5^ cells/well in 24-well plates before MccE492 or control treatments (phosphate-buffered saline (PBS) or mock). MccE492 was added to each well using a final concentration of 0, 30, or 60 μg/mL, and the mixture was incubated for 24 h. After incubation, cells of each well were trypsinized, suspended in PBS, and divided into two cytometry tubes. The viability was measured by staining the cells with 30 nM of Sytox Green dead cell stain (Invitrogen, Catalog No. S34860) and incubated for 20 min at room temperature protected from light. After incubation, 50 μL of Liquid Counting Beads (BD, Catalog No. 335925) were added to quantify the number of cells. Viability assays were analyzed by flow cytometry using a FACScan (Becton Dickinson) with BD FACSDiva™ Software.

### Zebrafish Husbandry and Maintenance

Zebrafish (*Danio rerio*) embryos of the Tab5 strain were obtained by natural spawning according to Kimmel et al. (1995). Fertilized eggs were raised in Petri dishes at 28°C containing E3 medium (5 mM NaCl, 0.17 mM KCl, 0.33 mM CaCl_2_, 0.3 mM MgSO_4_, and 0.1 % methylene blue) on a 14 h:10 h light/dark cycle until transplantation. After introduction of human cells, embryos were grown at 34°C and in darkness, to avoid bleaching of stained human cells. All procedures were conducted following the recommendations described previously for the use of zebrafish in biomedical research (Cartner et al., 2020) and complied with the guidelines of the Animal Use Ethics Committee of the University of Chile and the Bioethics Advisory Committee of Fondecyt-Conicyt (funding agency).

### Zebrafish Xenograft Development

Prior to transplantation into zebrafish larvae, human cancer cells SW620 were stained with Vybrant™ DiD Cell-Labeling Solution (ThermoFisher) according to the manufacturer's instructions, in order to visualize and track them inside larvae. Stained cells were suspended in PBS at a final concentration of 50 cells/nL and loaded into borosilicate glass capillary needles. ~500 cells were injected into the perivitelline space (PVS) of 48 h post fertilization (hpf) embryos anesthetized with tricaine (0.02%, Sigma-Aldrich) and mounted in low-melting point agarose (1%). After injection, xenograft-harboring larvae were maintained at 34°C until the end of the experiment. To assure the homogeneity of the xenografts subjected to the different treatments, at 24 h post injection (hpi), successfully injected xenografts were selected choosing larvae displaying a tumor of similar size. After selection of the cohort, the tumors were photographed using an Olympus MVX10 stereomicroscope with a total magnification of 16,3X. Afterwards, larvae were individualized and maintained in 48-well plates until the end of the experiment.

### Intratumoral Microcin E492 Administration in Zebrafish Xenografts

Twenty four hours after transplantation of tumor cells, xenograft-harboring zebrafish larvae were anesthetized and mounted into an agarose layer. Then, larvae were injected with ~5 nl of 1 mg/ml mcce492 (equivalent to 5 ng), or the same volume of the mock purification or PBS. Then, larvae were dismounted, washed carefully to remove agarose remnants and tricaine, and then kept in E3 at 34°C. An average of 50 xenografts were injected per condition. this procedure was repeated 48 h after cell transplantation.

### Evaluation of Tumor Size in Zebrafish Xenografts

At 5 dpf, individualized larvae were anesthetized with tricaine [0.02% (w/v)] and mounted in a layer of low melting point agarose (1%), allowing the tumors to be photographed using an Olympus MVX10 stereomicroscope with a total magnification of 16.3X, as described previously (Varas et al., 2019). The tumor area was quantified in 3-dpf and 5-dpf larvae using Image J software. The relative tumor size (5 dpf/3 dpf) was calculated and plotted using GraphPad software.

### Statistical Analyses

Cell viability experiments conducted by flow cytometry were performed in biological duplicates. From the total event acquired, only singlets were counted, and the average percent population (P1, P2, and P3) was determined for each condition. Statistical significance of differences in cell populations size was assessed using a two-way ANOVA test with a Dunnet's post-test. Differences were considered significant with a *P* < 0.05.

For zebrafish xenograft experiments, the statistical significance of differences in tumor cell mass was determined using a two-way ANOVA test with a Dunnet's post-test. Differences were considered significant with a *P* < 0.05. The total number of individuals considered for each condition of this experiment was 50.

## Results and Discussion

### MccE492 Shows Toxicity Against HT29 and SW620 Human Colorectal Cancer Cells *in vitro*

First, we aimed to select suitable cancer cell lines to conduct the study and set up the zebrafish xenograft model. After reviewing the literature, the cell lines HT29 and SW620 were selected considering the following relevant features: (1) they are of human origin; (2) they are from colorectal cancer and thus originate from the intestine, the place in which this bacteriocin is likely to be produced by enteric bacteria; and (3) they have been successfully used in xenograft transplantation experiments with zebrafish, as reported previously (Fior et al., 2017). Both cell lines were obtained directly from the European Collection of Authenticated Cell Cultures to perform the experiments described below. As a negative control we selected Human Embryonic Kidney 293 (HEK-293) cells, mainly because they have a non-tumoral origin (Graham et al., 1977; Brodaczewska et al., 2016) and because we found that these cells were unable to proliferate and form foci of growth after transplantation in zebrafish larvae (Supplementary Figure 1A).

We purified the MccE492 peptide from culture supernatants of *E. coli* cells transformed with the pMccE492 plasmid, which carries the gene cluster allowing the production and export of mature MccE492 (further details can be found in the Methods section). After the purification process, MccE492 was eluted in acetonitrile-water mixtures, which were then lyophilized to remove the solvent and to concentrate the peptide. Additionally, a control “mock” purification was prepared following the same steps than used for MccE492, but starting with cultures of *E. coli* cells transformed with a pMccE492 derivative lacking the *mceB* and *mceA* genes (encoding the MccE492 precursor and immunity protein, respectively), but carrying functional copies of the rest of the MccE492 cluster genes. By using this control, we aimed to eliminate any possible contribution of possible contaminant proteins present in the MccE492 preparation to the antitumorigenic effect to be studied. We evaluated the presence of MccE492 in the purified fractions (before and after lyophilization) monitoring the antibacterial activity against sensitive *E. coli* lawns, observed as the formation of growth inhibition halos (Figure 1B). MccE492 antibacterial activity was detected in 40% ACN fractions obtained from *E. coli* cells transformed with pMccE492, as well as in lyophilized preparations obtained from these fractions (suspended in PBS). Conversely, no antibacterial activity on MccE492 sensitive cells was observed with the purification obtained from the control strain. Also, we corroborated the presence of the MccE492 peptide by means of SDS-PAGE and immunoblotting using a monoclonal antibody prepared against this microcin. Although undetectable in the ACN fractions, a defined band corresponding to 10 kDa, and a more diffuse band between 15 and 20 kDa were observed in the lyophilized MccE492 preparation, but not in the mock control (Figure 1C). Although MccE492 has a molecular mass of ~8 kDa, it typically migrates anomalously in SDS-PAGE gels as a size corresponding to 10 kDa and also shows species with higher molecular mass that would correspond to SDS-resistant oligomers (Marcoleta et al., 2013a; Aguilera et al., 2016).

**Figure 2:**
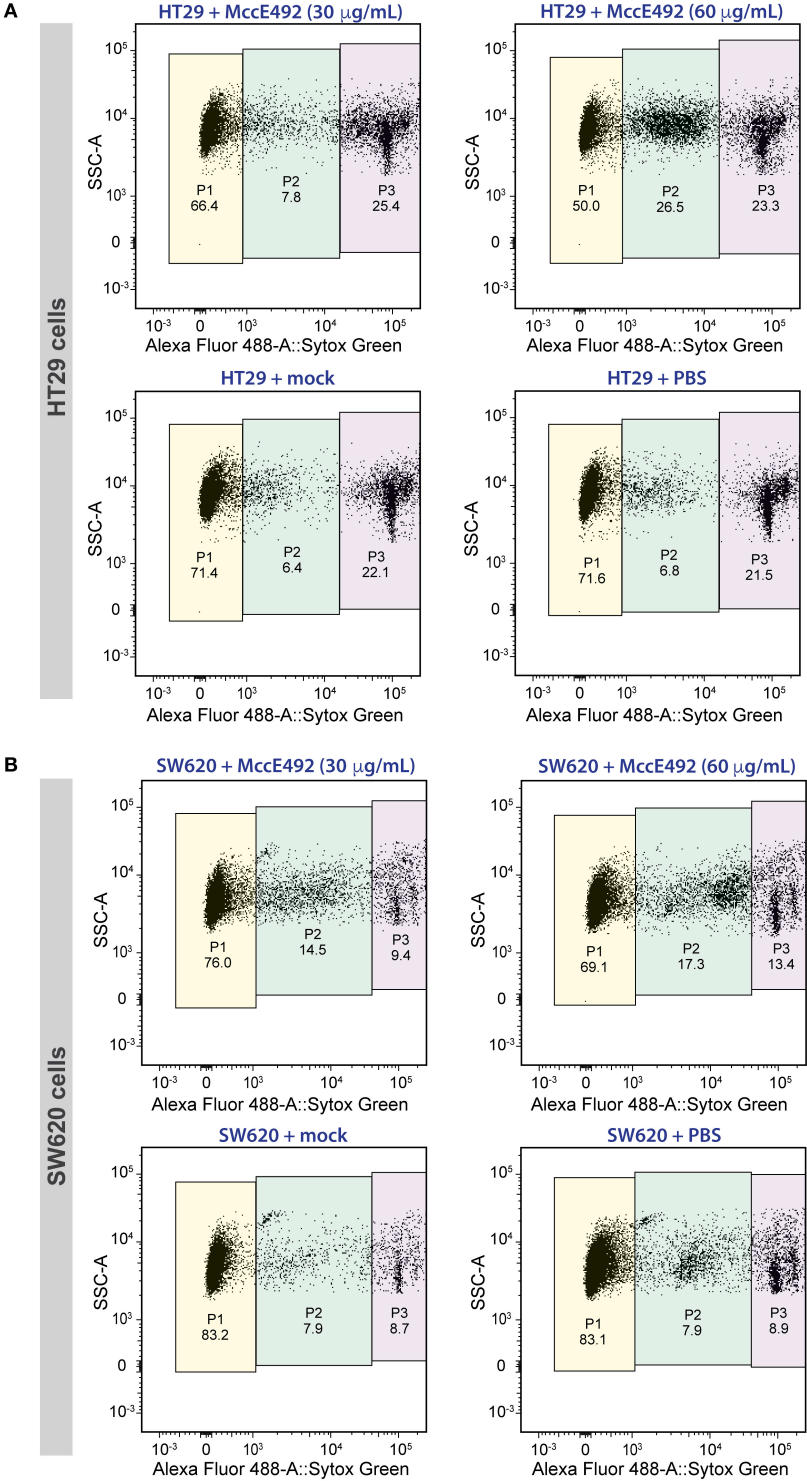
Microcin E492 induces cytotoxicity over human colorectal cancer cell lines *in vitro*. HT29 **(A)** and SW620 **(B)** cells were incubated during 24 h with MccE492, a mock purification, or PBS, and the cell viability was determined through flow cytometry using the Sytox Green Dead Cell stain (Invitrogen) and liquid counting beads. Colored areas denote the three populations distinguished (P1-P3), and the numbers under each population label indicate the percentage from the total population. Each plot shows a total of 10,000 cells.

Next, we evaluated the toxic activity of microcin E492 against the selected human colorectal cancer cell lines. To this end, 2.5 × 10^5^ cells of each line were seeded per well in 24-well plates. After 24 h, they were treated with 0 (PBS control), 30, or 60 μg/mL of MccE492, or with the mock purification for a period of 24 h. After incubation, cell viability was measured by flow cytometry using the Sytox Green Dead Cell Stain (Invitrogen) and Liquid Counting Beads (BD) for an accurate determination of the number of cells. For HT29 cells (Figure 2A), in absence of treatment (PBS) or upon mock treatment, the viable cell population (defined as P1) was around 71%. Two additional populations were observed, corresponding to dead cells (P3), accounting for 22–22.5% of the total population, and cells with compromised viability (P2), probably including mainly apoptotic cells, corresponding to 6.3–6.8%. MccE492 caused a marked dose-dependent decrease in cancer cell viability, which dropped to 66.4 and 50% upon treatment with 30 or 60 μg/mL microcin suspensions, respectively. Moreover, this effect was accompanied by a significant increase mainly in the P2 population (*p* < 0.001, Two-way ANOVA with Dunnet's multiple comparison test), especially after treatment with the higher MccE492 dose, where compromised cells increased to 26.5% of the total population. This indicates that MccE492 has a strong cytotoxic effect on HT29 cells *in vitro*, likely inducing an apoptotic response. As expected, MccE492 did not present a cytotoxic effect on an *in vitro* culture of HEK-293 cells (Supplementary Figure 1B).

A less marked effect was observed with the SW620 line (Figure 2B). Viability of untreated cells and cells subjected to mock treatment was around 83% (P1), showing a less marked reduction to 76 and 69% upon treatment with 30 or 60 μg/mL MccE492, respectively. As observed for HT29, this decrease was concomitant with an increase mainly in P2 cells (*p* < 0.05), and to a lesser extent, with the increment of dead cells (P3). Although SW620 cells presented a higher resistance to the MccE492 effect when compared to HT29 cells, both of them showed significant sensitivity to the toxic effect of this bacteriocin, supporting the observations made previously using other cancer cell lines like HeLa, Jurkat, and RJ2.25 (Hetz et al., 2002). The apoptotic effect of MccE492 has only been characterized previously in HeLa cells, and this phenomenon was associated with the release of calcium from intracellular stores, probably triggered after MccE492 pore-formation either in the mitochondria or in the cell membrane (Hetz et al., 2002).

It has been reported that the SW480 cell line, which is related to SW620, is sensitive to nisin, another bacteriocin (Ahmadi et al., 2017). The exposure to 1–4 mg/mL of nisin had a cytotoxic effect over SW480 colon cancer cells. The SW480 line was derived from the same patient as SW620 (used in this study), but the former proceeds from the primary tumor while SW620 was isolated 6 months later from a lymph node metastasis (Hewitt et al., 2000). Additional evidence indicated that nisin induced apoptosis in head and neck squamous cell carcinoma, as evidenced by an increase of intracellular calcium concentration, cell cycle arrest, and increased CHAC1 activation, a cation transporter and pro-apoptotic component of the unfolded protein response (Joo et al., 2012). Of note, another study reported that roughly 100 μM (~300 μg/mL) nisin had a strong cytotoxic effect over Caco-2 and HT29 colorectal cancer cell lines (Maher and McClean, 2006). This is a significantly lower dose, as compared to the IC50 reported for SW480 (Ahmadi et al., 2017). In agreement with these results, we also observed decreased susceptibility of SW620 cells to the bacteriocin-mediated cytotoxic effect, compared to HT29 cells. Additionally, the bacteriocin enterocin-B produced by *Enterococcus faecium* exhibited cytotoxic activity against HeLa, HT29, and other cancer cell lines, causing hallmark apoptotic morphological changes such as membrane blebbing, smaller nuclei, apoptotic body formation and nuclear fragmentation (Ankaiah et al., 2018).

Another antimicrobial peptide targeting the membrane showing anti-cancer activity is KL15, designed *in silico* based on the sequences of bacteriocins m2163 and m2386 from *Lactobacillus casei* ATCC 334 (Chen et al., 2015). KL15 displayed an anti-proliferative effect on SW480 cells inducing morphological changes and increasing membrane permeability. Also in this case, a limited pro-apoptotic effect was observed in these cells, further evidencing the reduced sensitivity of the SW480 and SW620 cell lines to pro-apoptotic bacteriocins. In this regard, Chen et al. (2015) proposed that KL15-mediated cytotoxicity over SW480 cells could be exerted through the activation of a non-apoptotic cell death pathway or by regulated necrosis (Berghe et al., 2014) involving hyperactivation of poly(ADP-ribose) polymerase (PARP-1), mitochondrial permeability changes, and formation of necrosomes and inflammasomes. An additional example of bacteriocins with cytotoxic activity over human cancer cells *in vitro* is a pediocin produced by *Pediococcus acidilactici* K2a2-3, which caused a ~55% growth inhibition of HT29 human colon adenocarcinoma cells (Villarante et al., 2011). Also, the 6.2-kDa Enterococcal anti-proliferative peptide (Entap) showed antiproliferative activity against cell lines of human gastric adenocarcinoma (AGS) and colorectal adenocarcinoma (HT29) (reviewed in Karpiński and Adamczak, 2018).

### Intratumoral Injection of MccE492 Reduces Tumor Growth in Zebrafish Xenografts of SW620 Human Colorectal Cancer Cells

**Figure 3:**
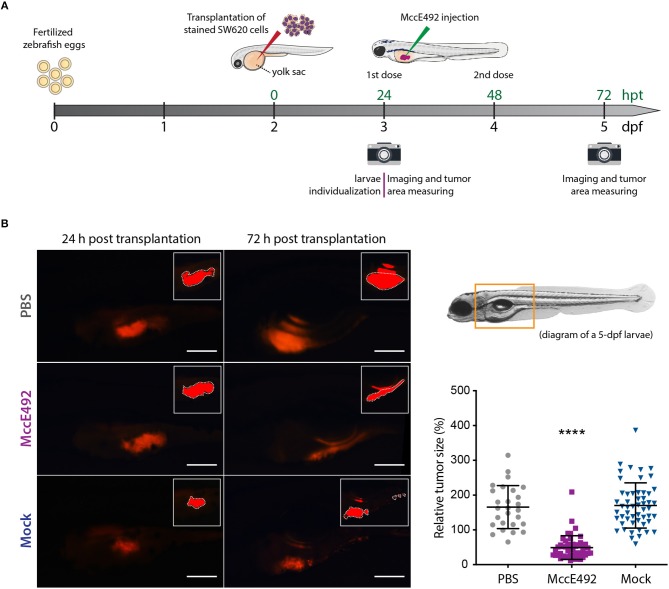
Microcin E492 intratumoral injection reduces the tumor size in zebrafish larvae SW620 xenograft model. **(A)** Schematic representation of the experimental setup for the development of zebrafish xenografts using SW620 human colorectal cancer cells to test the MccE492 antitumoral effect. **(B)** Assessment of the tumor cell mass size through live-cell imaging in zebrafish xenografts upon injection of MccE492, a mock purification, or PBS. Representative images of each condition are shown in the left side. The photographed area is delineated in the 5-dpf larva scheme shown. The insets show the saturated fluorescence area used in the estimation of tumors size (only the zones inside the white dashed lines were considered, as the excluded red zones correspond to reflections of the fluorescence signal in the swim bladder). The relative tumor size, corresponding to the percent change in tumor size comparing the same individual at 24 and 72 hpt, was determined and plotted for a total cohort of 50 individuals per condition. Error bars correspond to the standard deviation. ****P < 0.0001 (two-way ANOVA with a Dunnet's post-test).

Upon corroborating the cytoxicity of MccE49 over the tested cancer colorectal cells *in vitro*, we attempted to use them to develop a xenograft model using zebrafish larvae. Initially, both the HT29 and SW620 cell lines were explored for this purpose. However, in our hands, the transplantation of HT29 cells was less successful, as we could not achieve the xenografts efficiently in a large number of individuals. Thus, we decided to focus our efforts in developing SW620 xenografts, establishing the experimental set up schematized in Figure 3A. Prior to the transplantation, SW620 cells were fluorescently labeled using the Vybrant™ DiD Cell-Labeling Solution (ThermoFisher), in order to track cancer cells and tumor development inside larvae by live-cell imaging. ~500 cells were transplanted into individualized 2-dpf zebrafish larvae through injection into the perivitelline space, maintaining xenograft-bearing larvae at 34°C throughout the whole experiment. Twenty four hours post transplantation (hpt), the developing xenograft tumor was photographed to estimate its size using a fluorescence stereomicroscope, as described in the Methods section. Immediately after, either MccE492 (5 nL of 1 mg/mL solution in PBS) or an equivalent volume of the mock purification or PBS (used as MccE492 vehicle) were injected into the xenograft, and a second dose was applied at 48 hpt. Finally, at 72 hpt a second photograph of the xenograft was obtained and the relative tumor size was estimated (the tumor size measured at 72 hpt divided by the tumor size of the same individual at 24 hpt). This temporal frame was selected mainly because after 72 hpt, transplanted cells significantly dilute the fluorescent label due to proliferation, and zebrafish larvae progressively become less transparent, hampering an accurate evaluation of the tumor size.

As shown in Figure 3B, the analysis of a total cohort of 50 individuals from at least three biological replicates of the entire assay, indicated that MccE492 treatment -but not the mock treatment- significantly reduced the tumor size (*P* < 0.0001), compared to the group that received the PBS injection. Pictures of representative larvae from each condition are shown, in which the insets correspond to the saturated fluorescence area considered to accurately estimate the tumor size, as described previously (Zhao et al., 2011; Fior et al., 2017; Avci et al., 2018). The zone of the larvae shown in the pictures corresponds to that marked in the 5-dpf larva diagram. Our results indicate that MccE492 has a strong antitumoral activity on human colorectal SW620 cancer cells *in vivo*, supporting its potential use as an anti-cancer agent. Moreover, upon measuring the survival after 8 dpf of 50 xenografts per condition injected with either MccE492 or PBS, no significant changes were observed (98 and 92%, respectively), supporting that MccE492 has not a deleterious effect on larvae at longer times.

Previous works, by several research groups, point out the high potential of zebrafish larvae to carry out drug screening and for disease modeling (reviewed in Lu et al., 2017). As used here, larvae can be kept singly in multi-well plates allowing us to track the temporal evolution of transplanted human cancer cells in each individual through live-cell imaging. This set up allows simultaneous multiple non-invasive evaluations. Also, the reduced volume of the wells containing the larvae during the assays results advantageous when the drug availability is limited or in large-scale assays involving several conditions and numerous cohorts, where the total amount and number of compounds to be tested multiplies. In particular, the zebrafish xenograft approach has been especially exploited to study the behavior of human colon cancer cell lines and in colorectal tumor cells derived from patients (reviewed in Lobert et al., 2016). In a previous study, labeled human colorectal cancer cells of the lines SW620 and SW480 were injected into the yolk sac or perivitelline space of 2-dpf zebrafish embryos. The cancer cells then proliferated, migrated, and formed compact masses near the intestinal lumen surface (Haldi et al., 2006). Moreover, it was shown that the relative metastatic capacity of colorectal cancer cell lines *in vitro* and in *in vivo* mouse models is recapitulated in zebrafish. Likewise, SW620 cells reported as highly invasive on *in vitro* transwell migration assays were found to disseminate widely in zebrafish larvae a few days after transplantation (Teng et al., 2013). Conversely, HT29 cells that are not invasive *in vitro*, behave as non-metastatic inside larvae. Our results also indicate that the SW620 line is highly invasive, forming secondary tumors in a high number of individuals. The correlation between the known metastatic potential of cancer cells and their *in vivo* behavior upon transplantation into zebrafish allows (1) the evaluation of the metastatic potential of primary patient-derived tumors, and (2) the testing of compounds that will potentially reduce the invasiveness of these cells.

As a highly promising contribution in the field of personalized diagnostics and medicine, zebrafish xenograft models can be used for the evaluation of customized anti-cancer drug schemes. In this context, primary patient-derived biopsy specimens, often difficult to grow *in vitro*, can be used to isolate cancer cells and to establish xenografts in zebrafish, which after a few days are ready for testing the response of the cells to different anti-cancer agents. This is critical considering that currently patients go through multiple rounds of trial-and-error approaches to find the best treatment, with the consequence of adverse effects without therapeutic improvement, as well as the loss of valuable time before starting an effective treatment. In this sense, a recent study reported that colorectal cancer xenografts developed in zebrafish, starting from resected tumor samples from patients, constitute an advantageous *in vivo* model for testing differential therapy responses (Fior et al., 2017). The authors directly compared xenografts developed in zebrafish and in mouse, observing a good correlation between the relative sensitivities to anti-cancer agents in the two models. Also, several cell lines were tested in parallel including the closely related SW480 and SW620. Remarkably, they observed distinct proliferation dynamics, metastatic potential, and response to therapy between the isogenic tumors formed by these lines, revealing differential responses between primary and metastatic tumors to the tested drugs.

In summary, our data support MccE492 as a new antitumorigenic compound produced by bacteria. This polypeptide presents multiple advantages: it is small and very stable, resistant to proteases and to harsh conditions including boiling (de Lorenzo, 1984; Lagos et al., 1993), being stability one of the main requirements for a compound with potential pharmacological use. The fact that it is produced by bacteria also provides the possibility for direct delivery through a therapeutic infection with a probiotic. Finally, using the zebrafish model with many different types of xenografted cancers broadens the possibilities of finding successful targets for MccE492 antitumoral activity.

## Data Availability Statement

All datasets generated for this study are included in the article/Supplementary Material.

## Ethics Statement

The animal study was reviewed and approved by Comité de Etica, Facultad de Ciencias, Universidad de Chile.

## Author Contributions

MV, AM, MA, and RL conceived this study. MV and VS designed and performed the flow cytometry experiments and analyzed the data. MV, CM-M, VK, and AM performed the experiments with zebrafish larvae. AM, MV, and RL wrote the manuscript. All authors contributed with valuable discussions and edition, approving the final version of manuscript.

### Conflict of Interest

The authors declare that the research was conducted in the absence of any commercial or financial relationships that could be construed as a potential conflict of interest.
