# Supplementary material for: Exploiting Zebrafish Xenografts for Testing the in vivo Antitumorigenic Activity of Microcin E492 Against Human Colorectal Cancer Cells
Source: Front Microbiol. 2020 Mar 19;11:405. doi: 10.3389/fmicb.2020.00405 (PMC7096547; doi:10.3389/fmicb.2020.00405)
Supplement: Supplementary file 1 [file Image_1.pdf]

Exploiting zebrafish xenografts for testing the *in vivo* antitumorigenic activity of microcin E492 against human colorectal cancer cells

Macarena A. Varas[1], Carlos Muñoz-Montecinos[2], Violeta Kallens[2], Valeska Simon[3], Miguel L. Allende[2], Andrés E. Marcoleta[1,*], Rosalba Lagos[1,*].

Supplementary Material

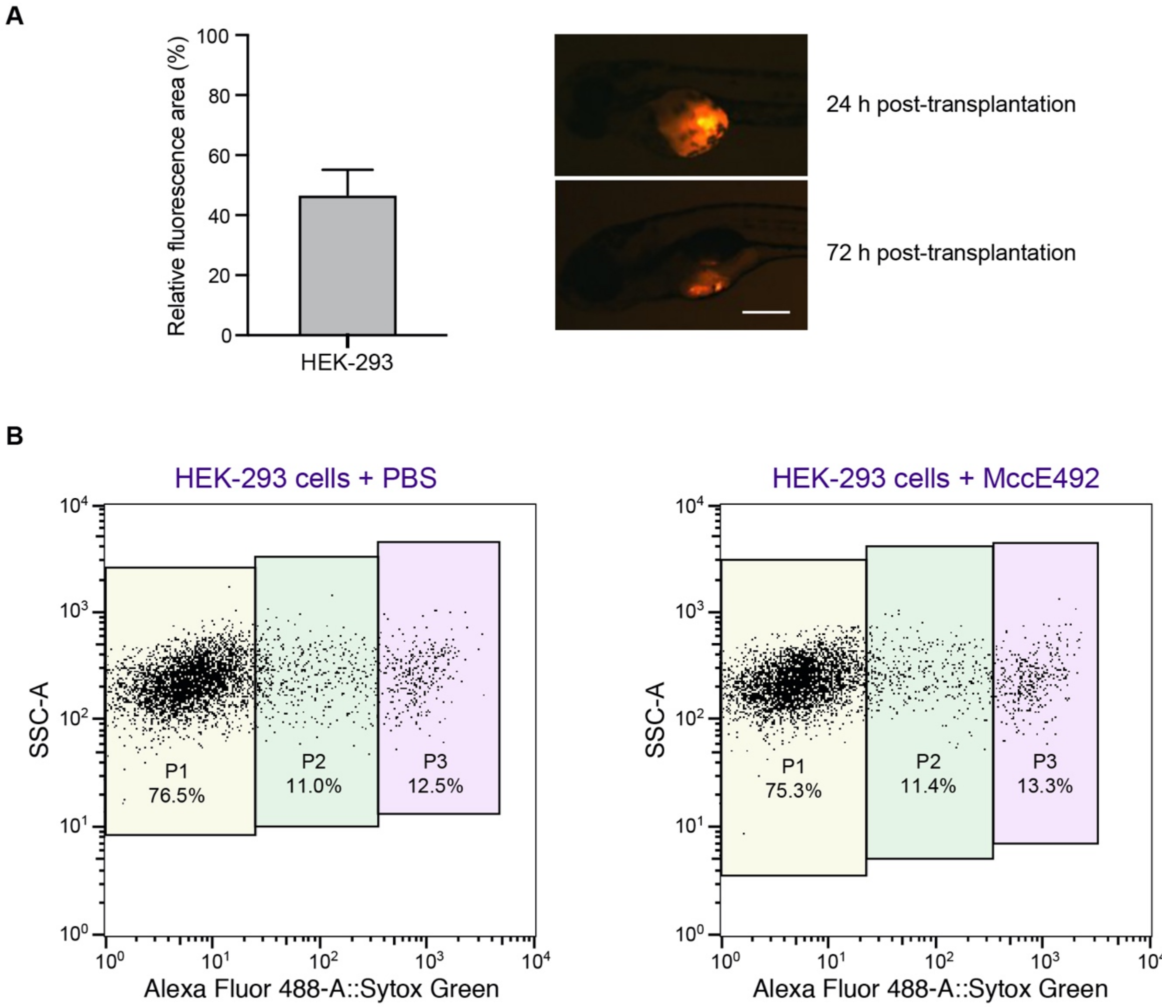

Supplementary Figure S1. Microcin E492 has no cytotoxic effect over HEK-293 cells, which do not form proliferative tumor masses in zebrafish larvae. A: Relative fluorescence area registered at 72 h post-tranplantation, as compared with 24 h post-tranplantation. The error bar represent the average and the standard deviation from 19 larvae. Scale bar: 100 μm. B: Flow cytometry-based viability determination of HEK-293 cells treated with purified MccE492 or with PBS. The yellow areas indicates viable cells (P1), green areas indicates apoptotic cells (P2), and purple area indicates dead cells (P3).
